# Deiodinase Types 1 and 3 and Proinflammatory Cytokine Values May Discriminate Depressive Disorder Patients from Healthy Controls

**DOI:** 10.3390/jcm12196163

**Published:** 2023-09-24

**Authors:** Elżbieta Małujło-Balcerska, Tadeusz Pietras

**Affiliations:** 1 Department of Pneumology, Medical University of Łódź, 90-419 Łódź, Poland; 2 Department of Clinical Pharmacology, Medical University of Łódź, 90-419 Łódź, Poland; tadeusz.pietras@umed.lodz.pl; 3 Second Department of Psychiatry, Institute of Psychiatry and Neurology, 02-957 Warsaw, Poland

**Keywords:** iodothyronine deiodinase, cytokine, depressive disorder, biomarkers

## Abstract

Introduction: Depressive disorders are multifactorial diseases in that a variety of factors may play a role in their etiology, including inflammation and abnormalities in the thyroid hormone (TH) metabolism and levels. The purpose of this study was to evaluate iodothyronine deiodinases (DIOs) and DIO-interacting cytokines as possible biomarkers in the diagnosis of depressive disorders. Methods: This study enrolled 73 patients diagnosed with recurrent depressive disorder (rDD) and 54 controls. The expressions of *DIO1*, *DIO2*, *DIO3*, *IL1B*, *IL6*, *TNFA*, and *IFNG* genes, encoding three types of DIOs (1, 2, and 3), interleukin (IL)-1β, IL-6, tumor necrosis factor (TNF)-α, and interferon (IFN)-γ, were assessed using the polymerase chain reaction in blood cells and an enzymatic immunoassay method in serum. The levels of examined molecules between patients and controls were compared, and correlations and diagnostic values were evaluated. Results: Lower levels of *DIO2* and higher levels of *IL1B*, *IL6*, and *TNFA* were found in patients compared to controls. The protein concentrations of DIO1 and DIO2 were lower, while that of DIO3 was higher, in patients than in controls. Serum IL-1β, IL-6, and TNF-α were also higher in patients than in controls. The area under the curve (AUC) of the IL-1β, IL-6, DIO1, and DIO3 proteins was >0.7 for discriminating patients with rDD from controls. Conclusions: The expressions of genes for DIO2, IL-1β, IL-6, and TNF-α may have a role in the estimation of processes present in depressive disorders. We can cautiously claim that DIO1 and DIO3 and pivotal cytokines, mainly IL-1β and IL-6, may play a role in depression diagnosis, and further studies are suggested to explain the exact role of these molecules in larger samples with more precise methods.

## 1. Introduction

Depressive disorder is an often complex psychiatric disorder. The identification of objective biomarkers that may support depressive disorder diagnosis is a very important issue [1]. The etiology of depressive disorder is multifactorial, with abnormalities in neurotransmitter systems [2], the presence of inflammation [3], and disturbances in thyroid hormone (TH) levels [4].

The involvement of inflammation and proinflammatory cytokines in the etiology of depressive disorder has been widely investigated [3]. There is evidence that depressive disorder is characterized by increased activity of immune cells and increased levels of proinflammatory cytokines, especially pivotal cytokines such as interleukin (IL)-1β, IL-6, tumor necrosis factor (TNF)-α, and interferon (IFN)-γ [3]. The concentrations of proinflammatory cytokines (TNF-α and IL-2) have been found to correlate with depression severity [5]. High concentrations of IL-1β and TNF-α have recently been observed to be related to the early onset of depressive disorder [6]. Changes in IL-6 concentration have been found to be associated with the effectiveness of antidepressant treatment, including widely used antidepressant drugs [7] and the newly used ketamine [8]. IFN-γ may play a role in reducing L-tryptophan availability, which is an important source of serotonin, a key neurotransmitter [9].

The coexistence of thyroid-related abnormalities with depressive disorder has been found. The observed abnormalities include changes in the reaction of thyroid-stimulating hormone (TSH) to thyrotropin-releasing hormone (TRH), the downregulation of TRH, higher levels of TRH in the cerebrospinal fluid (CSF), high concentrations of T4 in the periphery, and reverse triiodothyronine (T3) levels in CSF [10,11,12]. T3 has been found to have antidepressant effects in humans [13]. Animal model studies have revealed higher concentrations of T3 in the brain after the use of paroxetine, venlafaxine, and tianeptine, which are widely used antidepressants [14]. Disturbances in the levels of TH may not only be related to thyroid disorder. Low T3 syndrome is a nonthyroidal-related abnormality and is not related to increased TSH [15]. Studies have shown that some patients with depressive disorder are characterized by low T3, but the etiology is not related to severe illness, and a nonthyroidal illness is often involved [16].

The action and metabolism of THs involve many factors, including the hypothalamus–pituitary–thyroid axis, TH transporters that affect the influx of THs into the cells, and iodothyronine deiodinase (DIO), which regulates the metabolism of TH, while the signaling pathway of THs involves nuclear receptor and nongenomic pathways, such as membrane integrin [17]. The transporters, metabolizing enzymes, and receptors for THs are expressed in multiple brain areas, especially in the limbic system, and are known for their important functions, particularly those related to the pathomechanisms of depression [18]. Similarly, transporters for THs and all three types of DIOs have been expressed in immune cells.

Iodothyronine deiodinase (DIO) types 1, 2, and 3 are a group of selenoenzymes involved in the synthesis and determination of the levels of THs, including thyroxine (T4), T3, and reverse (r)T3 [17]. DIOs are tissue-specific. Type 1 deiodinase is mainly involved in the periphery; DIO2 is more important for brain-specific processes, while DIO3 is involved in brain-related and somatic physiology and pathology [19,20]. DIO1 tissue expression is observed in the thyroid gland, liver, and kidney; DIO2 expression is observed in the pituitary, skin, and brain; and DIO3 is mostly expressed in the central nervous system [21,22,23]. DIO1 and DIO3 have also been found in human circulating neutrophils, while the presence of DIO2 in monocytes has been widely described by Wenzek et al. [19].

Evidence has demonstrated that DIOs interact with both the immune system and the nervous system and are the link between TH-related factors and the immune system [19,20]. DIOs have been investigated as immune inflammatory-related molecules. DIO1 expression has been found to change during inflammation [24]. Cytokines such as interleukin (IL)-1β and IL-6 have been found to attenuate *DIO1* expression [25,26]. An increase in DIO2 due to inflammatory stimuli has been observed [27]. DIO3 plays an essential role in proper neutrophil activity during sterile and bacterial inflammation [28,29]. In the nervous system, DIOs determine TH levels and affect processes such as synaptic transmission, neurogenesis, neurodevelopment, and plasticity [30,31,32].

Therefore, DIOs not only participate in the determination of TH levels but may also play a role as markers of the inflammatory process and could be potential biomarkers of some inflammatory diseases.

Gene and protein expression levels may be appropriate biomarkers for psychiatric disorders, including depression, as has been presented in other studies [33,34].

This study used peripheral blood cells to estimate gene expression. According to Metha et al. [35], the similarity in the transcriptome between peripheral blood cells and other tissues, including the brain, is approximately 80%. The evaluated expression may resemble the expression levels in the periphery and brain. To measure protein concentrations, we used enzyme-linked immunosorbent assay (ELISA), a known method used to evaluate protein concentration. Cytokines are often examined using these methods. DIOs are tissue-specific enzymes but are also expressed in immune cells, and the serum levels of DIOs may be immune cell-derived.

The present study evaluated the peripheral blood gene expression of three types of deiodinases, iodothyronine, and pivotal cytokines, and their corresponding serum protein levels in patients suffering from depressive disorder. The main purpose was to explore whether the investigated molecules warrant further examination and might be explored as possible biomarkers for depression diagnosis and in determining the risk of depressive disorder development.

## 2. Materials and Methods

### 2.1. Participants

A total of 127 participants were recruited for this study: 73 rDD patients and 54 controls. Patients and controls were age- and sex-matched. The inclusion process was carried out by a qualified psychiatrist. All patients were examined during hospitalization. Patients with a diagnosis of rDD were screened in accordance with ICD-10 criteria [36]. The data concerning the course of the depressive disorder were collected based on the composite international diagnostic interview (CIDI) [37]. The number of depressive episodes, number of hospitalizations, and duration of the disease were collected for each patient. The Hamilton depression rating scale (HAM-D) was used to assess depressive disorder severity [38]. The exclusion criteria were as follows: the presence of psychiatric diseases other than depressive disorder, immunological diseases, acute or chronic infection, thyroid diseases, substance abuse, and treatment with anti-inflammatory agents. All the study participants were of Polish origin. The study was approved by the ethics committee and was conducted based on the principles of the Declaration of Helsinki. Written informed consent was obtained from all the participants. Table 1 presents the baseline characteristics of the enrolled participants.

### 2.2. Sample Collection and Estimation of Gene Expression and Serum Concentration

Blood was collected from the participants via the vein between 7 and 8 a.m. into tubes with EDTA anticoagulant and without anticoagulant to obtain sera. Samples for mRNA evaluation were stored at −70 °C. Serum was separated from peripheral blood using clot-activating tubes. Next, the samples were allowed to clot for 30 min and then centrifuged for 15 min at 1000× *g*. The serum was removed and stored as an aliquot. Serum samples were also stored at −70 °C until the experiments.

### 2.3. Detection of Gene Expression Using Real-Time PCR

Total RNA was isolated from the blood of all the participants using the modified Chomczyński method [39]. The concentration of the isolated RNA was assessed using a Picodrop spectrophotometer (Hinxton, United Kingdom) at λ = 260 nm. Next, the quality of RNA was verified according to the Agilent RNA 600 Nano Kit manual (Agilent Technologies, Santa Clara, CA USA). The obtained RNA was converted into cDNA via reverse transcription (RT) using the TaqMan^®^ RNA Reverse Transcription Kit (Applied Biosystems) based on the manual provided by the manufacturer. The conditions of the RT reaction were as follows: incubation (30 min, 16 °C, and 30 min, 42 °C) and inactivation (5 min, 85 °C) in a thermocycler (Biometra, Gottingen, Germany). After the RT reactions, cDNA was stored in the freezer at −20 °C.

To assess the mRNA expression levels of the examined genes, real-time PCR was performed in 96-well plates with TaqMan^®^ Universal PCR Master Mix, No UNG, and the following probes: Hs00174944_m1 for *DIO1*, Hs00988260_m1 for *DIO2*, Hs00956431_s1 for *DIO3*, Hs00174092_ml for *IL1B*, Hs 00985639_ml for *IL6,* Hs01113624_gl for *TNFA,* Hs00989291_m1 for *IFNG*, Hs04194366_g1 for *RPL13A,* and Hs04194366_g1 for 18S *rRNA* (Applied Biosystems, Foster City, CA, USA). The real-time PCRs were carried out under the following conditions: incubation at 50 °C for 2 min and 95 °C for 10 min, and then cyclization at 95 °C for 30 s, 60 °C for 30 s, and 72 °C for 1 min; 40 cycles were performed in total. The quantification of gene expression was performed with the use of the critical threshold (Ct) value. To calculate the expression of the examined genes at the mRNA level, the Ct comparative method was used [27], and ΔCt was determined (ΔCt = Ct analyzed gene-Ct reference gene). The results were analyzed according to the 2^−∆ct^ method [40]. To normalize the gene expressions of *DIO1*, *DIO2*, *DIO3*, *IL1B*, *IL6*, and *IFNG*, the *RPL13A* gene was used as a reference gene. To normalize the expression of *TNFA*, the *18S RNA* reference gene was used. The experiments were carried out with an ABI Prism 7000 (SDS Software, Applied Biosystem). For every assay, controls without RT and no template cDNA were performed.

### 2.4. Measurement of the Levels of the Iodothyronine Deiodinases and Cytokine Proteins with an Enzyme-Linked Immunosorbent Assay (ELISA)

Protein concentrations were evaluated using ELISA provided by R&D Systems, Inc. (MIN USA; DLB50, D6050, DTA00C, and DIF50 for IL-1β, IL-6, TNF-α, and IFN-γ, respectively) and MyBiosource (San Diego, CA, USA; MBS05509, MBS039426, and MBS108556, for human DIO1, DIO2, and DIO3, respectively). The analysis and calculations were performed based on protocols supplied by the manufacturers. A Multiskan Ascent Microplate Photometer (Thermo Labsystem, Waltham, MA, USA) was used to measure the absorbance of the sample at λ = 450 nm. To determine the protein concentration, an analytical curve was generated. The concentrations of IL-1β, IL-6, TNF-α, and IFN-γ are expressed in pg/mL, and the concentrations of DIO1, DIO2, and DIO3 are expressed in international units (U/L).

### 2.5. Statistical Analysis

The data were analyzed with Statistica (version v13.1; Tibco). The data are presented as the mean ± standard deviation, and *p* < 0.05 was considered statistically significant. A normal distribution of continuous variables was found with the one-sample Kolmogorov—Smirnov test. The comparison of the age and dependent variables between investigated groups was performed using the Student’s *t*-test for independent samples or the Student’s *t*-test with an independent estimation of variance when there was no homogeneity of variance. Effect sizes were determined using Cohen’s d, which was characterized as large (d > 0.8), moderate (d between 0.8 and 0.5), small (d between 0.49 and 0.20), or trivial (d < 0.05) for pairwise comparisons. The sex difference between the groups was compared using the chi-square test. Pearson correlation tests were used to perform correlation analyses. A logistic regression model was used to predict the outcome of dependent variables. The Hosmer—Lemeshow goodness-of-fit test for logistic regression was performed. To evaluate the area under the curve (AUC) of variables for discriminating the rDD group, receiver operating characteristic (ROC) curve analysis was used.

## 3. Results

Baseline characteristics of the participants are shown in Table 1.

Levels of cytokines and iodothyronine deiodinase gene expression and protein concentrations in rDD patients and controls are presented in Table 2.

Higher levels of *IL1B*, *IL6*, and *TNFA* mRNA and the corresponding proteins were observed in enrolled patients than in controls. No significant differences between the investigated groups in the mRNA and protein levels of *IFNG* and IFN-γ were observed. The mRNA of *DIO2* and DIO2 protein levels was significantly decreased in the rDD patients compared to the controls. Differences in the protein levels of DIO1 and DIO3 between the investigated groups were found, with significantly lower levels of DIO1 and significantly higher levels of DIO3 in the rDD group than in the control group.

Results of correlation and logistic regression analysis.

A significant relationship was found between the levels of DIO2 mRNA and disease duration (r = −0.34, *p* = 0.01) and between DIO3 and the number of episodes (r = 0.27, *p* = 0.02). Correlation analysis also found a negative correlation between *DIO2* expression and *IL6* (r = −0.81, *p* < 0.001) and between *DIO2* and *TNFA* (r = −0.83, *p* < 0.001).

Binary logistic regression analysis revealed that an increase in IL-1β (OR: 22.81, 95% CI 1.40–4.86, *p* < 0.001) and IL-6 (OR: 115.89, 95% CI 1.06–8.44, *p* = 0.012) proteins increased the risk of rDD; an increase in DIO1 protein (OR: 0.43, 95% CI −1.65–0.013, *p* = 0.046) reduced the risk of rDD by 2.3 times; and an increase in DIO3 protein (OR: 2.94, 95% CI 0.52–1.64, *p* < 0.001) increased the risk of rDD.

Diagnostic value in distinguishing patients with rDD from healthy controls.

**Figure 1:**
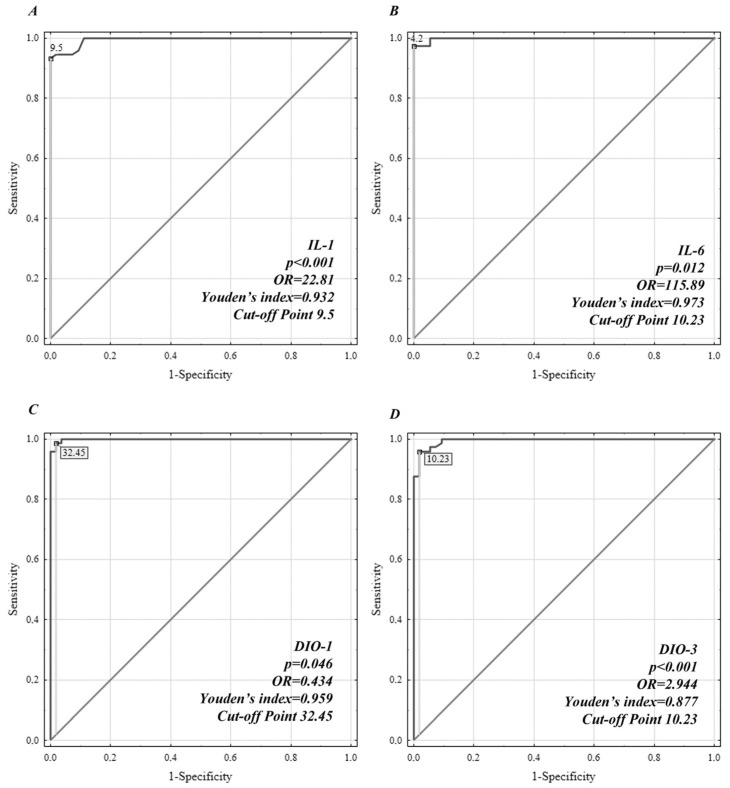
ROC analysis of interleukin (IL)-1β, IL-6, DIO1, and DIO3 protein concentrations for distinguishing patients with depressive disorder from healthy controls. (**A**) ROC of IL-1β, (**B**) ROC of IL-6, (**C**) ROC of DIO1, and (**D**) ROC of DIO3.

ROC curve analysis was performed to discriminate depressive patients from healthy controls, and the AUCs for IL-1β, IL-6, DIO1, and DIO3 proteins were >0.7, indicating diagnostic value (Figure 1). The AUC values were 0.995, 0.998, 0.99, and 0.995 for IL-1β, IL-6, DIO1, and DIO3, respectively.

For IL-1β, the sensitivity and specificity were 94.5 and 98.1, respectively, and the cut-off point was 9.4 pg/mL; for IL-6, the sensitivity and specificity were 97.3 and 100, respectively, and the cut-off point was 4.2 pg/mL; for DIO1, the sensitivity and specificity were 98% and 100%, respectively, and the cut-off point was 11.37 U/L; and for DIO3, the sensitivity and specificity were 95.9 and 98.1, respectively, and the cut-off point was 10.23 U/L.

## 4. Discussion

The present study compared four pivotal cytokines and three DIOs between patients diagnosed with depressive disorder and control subjects. This study is a further analysis of our previously published results [41,42], enlarging the analysis with data not used, with the main aim of determining the role of the examined markers as possible useful biomarkers in identifying the risk of developing rDD and in rDD diagnosis. First, the data revealed that compared to controls, patients had increased levels of *IL-1B*, *IL-6*, *TNF-*α, and *DIO3* mRNA and protein and decreased levels of *DIO2* mRNA and protein. Deiodinase type 1 expression differences between the groups were found only at the protein level. Second, correlations between the examined parameters were found. Third, we determined that the concentrations of IL-1β, IL-6, DIO1, and DIO3 may influence the development of depression. The AUC values >0.7 for IL-1β, IL-6, DIO1, and DIO3 suggest the involvement of these molecules as possible discriminating biomarkers between patients and controls. This last finding is an important novelty of this study.

Cytokines are important for cellular connections and signaling pathways [43]. Cytokines such as IL-1β, IL-6, TNF-α, and IFN-γ are known for their importance in depression mechanisms [44]. The results of our study are consistent with data presented by Rizavi et al. [45], who found that the mRNA levels of *IL-1B*, *IL-6*, and *TNF-*α were significantly higher in the blood cells of patients with depression than those of healthy controls. A number of studies have reported changes in the protein levels of cytokines in patients suffering from depressive disorder. The first study was published twenty years ago [46], and it has been reported recently that patients suffering from depressive disorder are characterized by increased concentrations of IL-1β [47], IL-6, and TNF-α [34,48] compared to controls. There was no indication of IFN-γ involvement in depression in our study. In contrast, Daria et al. [49] found lower IFN-γ levels in patients with depression who were not treated with antidepressant drugs, while IFN-γ together with brain-derived neurotrophic factor was observed to be a risk factor for depressive disorder by Chen et al. [50].

Nowadays, the importance of the type 1/type 2 cytokine hypothesis is reflected in scientific research considering an inflammation−depression association [51]. Signs of increased activation of both type 1 and type 2 cytokine-related pathways have been observed and described [52]. Data presented in our study support the type 1 cytokine hypothesis of depressive disorder. Regarding the type 2 cytokine, further studies are warranted, including the measurement of mRNA for type 2 cytokines such as IL-4, IL-10, protein concentrations, and the examination of the role of these biomarkers in the diagnosis of depressive disorder and in discriminating patients suffering with depressive disorder from healthy controls. In addition, more inflammatory markers are worth examining in depressive disorder. For example, the emerging role of macrophage migration inhibitory factors (MIF) in depressive disorder should not be omitted [53].

Thyroid hormones and TH-related factors, including DIOs, have been evaluated in previous preclinical and clinical studies on psychiatric disorders and inflammatory diseases by other research groups, producing different results that are discussed below. DIOs are important for the maintenance of proper TH concentration and function in the brain and other tissues and at low peripheral levels. Equilibrium in TH levels is important because, for example, excess THs related to a deficit of DIO3 result in behavioral abnormalities [54].

Thyroid disorder and hyper- and hypothyroidism often occur along with depressive disorder [55]. TH metabolism has been found to be influenced by alcohol addiction, which may increase the risk of depression [56]. DIO1 and DIO3 may participate in the mechanism of depression risk during alcohol consumption; after three months of reduced alcohol usage, DIO1 activity increased and reached the normal range, while DIO3 activity was inhibited [57]. Higher expressions of *DIO2* and *DIO3* in the brain hippocampus have been associated with higher resilience to stress-associated depression [58], while higher levels of DIO2 activity measured in brown tissue correlated with disruption in the quality of sleep [59], one of the features of depression [60]. In the group of neuro-related behavioral disorders with inflammatory-related mechanisms [61], autism spectrum disorder has been found to be related to lower *DIO2* expression [62]. An animal model study has found that DIO2 deficits in certain areas of the brain may cause mood behavioral disturbances, anxiety, and an increase in fear memory [63]. The activity of DIO1 may be regarded as a possible factor participating in the mechanism of antidepressant therapy, as some drugs, such as phenytoin and carbamazepine, induce DIO1 activity, possibly similar to its expression in patients with developmental disorders [64], a process related to depressive disorder [60].

In the present study, depressive patients exhibited lower levels of DIO1 and DIO2 proteins and higher levels of DIO3 protein, while *DIO2* mRNA expression and protein levels were lower.

Peripheral levels of different biomarkers, including DIOs, may reflect processes related to depression that occur in the periphery but may also reflect processes that occur in the brain.

There are limited data regarding the peripheral expression of *DIO1*/DIO1. *DIO2*/DIO2, and particularly *DIO3*/DIO3, have been found in peripheral cells during different kinds of inflammation, which may indicate the involvement of these selenoenzymes in inflammation-related disturbances. Regarding the inflammatory-related mechanism, the decrease in *DIO2* expression at the mRNA and protein levels in our study was inconsistent with studies that reported *DIO2* mRNA upregulation in macrophages during acute inflammation [27]. According to our results, a negative correlation between *DIO2* mRNA and *IL6* and *TNFA* mRNA was found, but no correlation was found at the protein level. *DIO2*/DIO2 gene and protein levels are influenced by many posttranscriptional and posttranslational processes and proteasome systems, which should be further explored. For example, TH may reduce protein levels and activity while not affecting mRNA levels [23], DIO2 may be inhibited by different factors [65], and antidepressant therapy might not be excluded. Lower levels of DIO2, an important predictor of thyroid hormone levels in the brain, may impact disturbances in brain-related processes often present in depression. A decrease in *DIO2* expression in mouse astrocytes correlated with anxiety and depressive-like behavior [66]. Lower levels of DIO2 may reflect the presence of proinflammatory processes, as the inhibition of DIO2 has been observed along with high proinflammatory effects expressed by an increase in inflammatory mediators, IL-1β, and COX-2 [67]. Deficiency in DIO2 expression results in the impaired death of bacteria [19], a known factor to induce depressive-like behavior [68]. In this case, the correlation between DIO2 mRNA and DIO3 concentration may cautiously support immune activity and the clinical outcome of depressive disorder. We did not find a correlation between DIO1 and cytokines, but further studies are warranted; the decrease in DIO1 levels in the periphery might be explained by proinflammatory stimuli, as they may reduce *DIO1* expression but increase *DIO3* expression. Changes in the expression of both factors may result in/from nonthyroidal syndrome that may coexist with depressive disorders [16]. DIO1 levels in the periphery may reflect the levels of THs that are linked to depressive disorder development and course [69] and may reflect the presence of inflammation, as low DIO1 has been found to be related to the presence of proinflammatory cytokines [26] and may reflect the number of monocytes, as the molecule has been expressed in this kind of blood cell [19]. DIO3 may reflect the peripheral and central levels of THs and the presence of inflammation, as increased levels of DIO3 have been characteristically found in inflammation [70], which needs further explanation. An increase in *DIO3* mRNA expression and protein concentration may determine blood immune cell (neutrophils) counts, as these cells express DIO3, which warrants further study. Different factors affect the levels of respective molecules, such as polymorphic variants, the abovementioned time-dependent expression of posttranscriptional and posttranslational modifications, and other molecules that can affect protein levels, ethnicity, pharmacotherapy, and even nutritional habits.

The serum concentrations of all types of DIOs may be immune cell-derived, and our study aimed to determine whether the examined cytokines and DIOs can be useful as biomarkers for the diagnosis of depressive disorder. For that purpose, ROC analysis was used. AUCs above 0.7 are known to be of clinical value, and our data revealed values of 0.995, 0.998, 0.99, and 0.995 for IL-1β, IL-6, DIO1, and DIO3, respectively.

The limitations of the study include the small sample size, the measurement of mRNA in blood cells and protein concentration in serum, the lack of measurement of TH concentration that would add value to this study, the influence of the housekeeping gene on the pathophysiology of depression, and the need for more precise methods.

## 5. Conclusions

This study cautiously suggests that an abnormal gene expression of *IL-1*, *IL6*, *TNFA*, and *DIO2*, as well as transcriptional measures within these genes, warrants consideration in further studies. Protein concentrations of IL-1β, IL-6, DIO1, and DIO3 may be suggested as potential diagnostic biomarkers in discriminating patients suffering from rDD from healthy controls with the enlargement and modification of the methods. Further studies are needed to confirm the results and increase the knowledge and potential usefulness of the abovementioned molecules as novel biomarkers for depressive disorder development.

The results of this study might help to estimate the risk of rDD using cytokines and iodothyronine deiodinase, separately or as a combined network. Moreover, data should be useful in further understanding the roles of cytokines and DIOs and might explain the disturbances in TH concentration in depression. The results should also help to answer whether the changes in the level of THs might be related to their metabolic pathway, a mechanism related to inflammation, or both. Considering the interactions among DIO1, DIO3, and inflammation, data may provide information on whether this relationship should be further investigated in depression in terms of inflammatory components of the disease. Positive results might be useful to identify and add to the groups of risk factors for depression appearance. In the case of positive results, analyses of protein levels, enzyme activity, and thyroid hormone levels should be planned. In addition, the data obtained on protein levels may be considered in further diagnostics and in planning pharmacotherapy.

## Figures and Tables

**Table 1:** Demographic characteristics of the rDD patients and controls.

	rDD n = 73	Controls n = 54	t/χ2	*p*
Rows	48.56 ± 5.23	48.50 ± 5.67	0.06	0.95
Sex (female/male)	34/39	25/29	0.001	0.98
Disease duration	5.30 ± 7.5			
Number of depressive episodes	3.32 ± 3.56			
Number of hospitalizations	2 ± 1.48			
Hamilton depression rating scale	23.53 ± 6.39			

rDD—recurrent depressive disorder; *p*—level of significance.

**Table 2:** Comparison of cytokine and DIO mRNA and protein levels between the rDD group and the control group.

Variable	rDD n = 73 (mean ± SD)	Controls n = 54 (mean ± SD)	t	*p*	Size Effect	Power
IL1 pg/mL	11.55 ± 1.44	6.81 ± 1.32	19.04	<0.0001	3.44	1.00
*IL1B* mRNA 2^−ΔCt^	0.70 ± 0.09	0.43 ± 0.08	13.82	<0.0001	3.20	1.00
IL-6 pg/ml	5.63 ± 0.89	2.11 ± 0.57	25.53	<0.0001	4.73	1.00
*IL6* mRNA 2^−ΔCt^	0.33 ± 0.06	0.12 ± 0.04	18.51	<0.0001	4.49	1.00
TNF-α pg/ml	11.08 ± 1.25	4.86 ± 1.02	29.95	<0.0001	5.46	1.00
*TNFA* mRNA 2^−ΔCt^	0.66 ± 0.07	0.29 ± 0.07	22.74	<0.0001	5.31	1.00
*IFNG* mRNA 2^−ΔCt^	0.17 ± 0.08	0.18 ± 0.07	−0.26	0.80		
IFN-γ pg/ml	4.97 ± 0.89	4.79 ± 0.81	1.16	0.25		
*DIO1* mRNA 2^−ΔCt^	0.07 ± 0.02	0.08 ± 0.01	−1.19	0.24		
*DIO2* mRNA 2^−ΔCt^	0.12 ± 0.2	0.32 ± 0.06	−20.55	<0.0001	4.21	1.00
*DIO3* mRNA 2^−ΔCt^	0.06 ± 0.01	0.06 ± 0.03	−1.63	0.11		
DIO1 U/L	21.21 ± 5.25	55.96 ± 11.88	−22.25	<0.0001	3.78	1.00
DIO2 U/L	13.58 ± 5.77	37.32 ± 4.51	−25.08	<0.0001	4.58	1.00
DIO3 U/L	15.75 ± 2.87	4.44 ± 2.46	23.32	<0.0001	4.23	1.00

## Data Availability

The data analyzed during this study are available from the corresponding author upon reasonable request.
